# Impact of the COVID-19 pandemic on management of children and adolescents with Type 1 diabetes

**DOI:** 10.1186/s12887-022-03189-2

**Published:** 2022-03-10

**Authors:** Abha Choudhary, Soumya Adhikari, Perrin C. White

**Affiliations:** Division of Pediatric Endocrinology, Department of Pediatrics, University of Texas Southwestern Medical Center, 5323 Harry Hines Boulevard, Dallas, TX 75390-9063 USA

**Keywords:** Continuous glucose monitor, Generalized linear model, Hemoglobin A1c, Patient health questionnaire -9, Telehealth

## Abstract

**Background:**

The coronavirus disease-2019 (COVID-19) pandemic had widespread impacts on the lives of parents and children. We determined how the pandemic affected Type 1 diabetes patients at a large urban pediatric teaching hospital.

**Methods:**

We compared patient characteristics, glycemic control, PHQ-9 depression screen, in person and virtual outpatient encounters, hospitalizations and continuous glucose monitor (CGM) utilization in approximately 1600 patients in 1 year periods preceding and following the local imposition of COVID-related restrictions on 3/15/2020 (“2019” and “2020” groups, respectively).

**Results:**

In a generalized linear model, increasing age, non-commercial insurance, Black and Hispanic race/ethnicity, and non-utilization of CGMs were all associated with higher hemoglobin A1c (HbA1c), but there was no difference between the 2019 and 2020 groups. The time in range in CGM users was lower with non-commercial insurance and in Black and Hispanic patients; it improved slightly from 2019 to 2020. CGM utilization by patients with non-commercial insurance (93% of such patients were in government programs, 7% uninsured or “other”) increased markedly. In 2020, patients with commercial insurance (i.e., private-pay or provided by an employer) had fewer office visits, but insurance status did not influence utilization of the virtual visit platform. There was no change in hospitalization frequency from 2019 to 2020 in either commercially or non-commercially insured patients, but patients with non-commercial insurance were hospitalized at markedly higher frequencies in both years. PHQ-9 scores were unchanged.

**Conclusions:**

Hospitalization frequency, glycemic control and depression screening were unchanged in our large urban pediatric teaching hospital during the COVID pandemic. Increased utilization of CGM and rapid adoption of telemedicine may have ameliorated the impact of the pandemic on disease management.

## Introduction

The coronavirus disease-2019 (COVID-19) pandemic disrupted daily life in many ways. In Texas, a state of emergency was declared on 3/13/2020 and a lockdown imposed on 3/21/2020. Non- essential businesses closed, schools were transitioned to virtual modes of education and most public activities ceased. Many studies have described the effects of parental employment, psycho-social stress [1–3] and access to health care [4, 5] on diabetes care. The pandemic may have impacted care for many children with diabetes through lack of access to school nurses, altered care arrangements, loss of parental jobs and insurance, social isolation and psychological stress, and clinic closures.

We retrospectively examined the impact of the pandemic on outcomes including hospitalization, clinic visits, glycemic control and the frequency of depression in children and adolescents with Type 1 diabetes (T1D) in our institution.

## Methods

### Setting

The clinical setting and database were previously described [6]. In brief, the study took place at Children’s Medical Center Dallas, a large urban pediatric teaching hospital licensed for 487 beds, with a 72-bed hospital also owned by Children’s Health System of Texas and staffed by the same endocrinology group located 22 mi (35 km) north. This study was approved by the UT Southwestern Institutional Review Board. Data were obtained using SAP (Walldorf, Germany) analytics to interrogate a Clarity database derived from our institutional Epic (Madison, WI) clinical data repository.

### Coding and statistical analysis

#### Study population (exposure)

Two sequential reports were created, a baseline “2019” pre-COVID group comprising encounters between 3/15/2019 and 3/14/2020 (the approximate day that COVID restrictions were imposed in Dallas County) and a “2020” post-COVID group representing encounters occurring between 3/15/2020 and 3/14/2021. Only patients with at least one outpatient encounter were included in either group.

We excluded patients with type 2 diabetes, genetic diabetes, cystic fibrosis-related diabetes or secondary (induced by corticosteroids or other drugs) diabetes, and patients with duration of diabetes < 365 days at the time of their most recent encounter.

#### Covariates

Age was assessed at the last visit of each year. Insurance status was coded as “commercial” (i.e., private-pay or provided by an employer, considered a marker for higher socioeconomic status [7]) or “non-commercial,” (93% of such patients were in government programs, 7% uninsured or “other”, considered to represent lower socioeconomic status). Race and ethnicity were recoded as a single variable with values of “White or Caucasian”, “Hispanic”, “Black or African American”, or “Other”; in our region, the vast majority of Hispanics are of Mexican origin. Patients were classified as continuous glucose monitor (CGM) users if any CGM downloads were present in the database for the given year.

#### Outcomes

Both inpatient admissions and observations, but not emergency department visits, with ICD-10 codes of E10.10, E10.11, and E10.65 were counted as hospital admissions. Number of office visits during the year was defined as the number of visits to our diabetes clinic with either a physician or a nurse practitioner, excluding education-only group classes. Virtual visits were defined similarly. Our clinic used an Alere Afinion Analyzer (Abbott Diagnostics, Lake Forest, IL) to measure hemoglobin A1c (HbA1c). We used the most recent HbA1c value in each year, which is the only one routinely routinely retained in the database. Values > 15 (i.e., above the linear range of the assay) were recoded as 15. For CGM users, the most recent two-week period available was used to assess time in range. Patients share their Dexcom CGM data with the clinic through the cloud- based Dexcom Clarity application. Freestyle Libre data were shared via Libreview. CGM metrics were documented in the electronic medical record at every visit. Time in range was defined as the proportion of CGM data points falling in the 70–180 mg/dL range. Patient Health Questionnaire-9 (PHQ-9), a self-administered depression screening was offered to all patients with T1D 10 years and older who were seen in person. It was offered every 9 months or sooner if there were mental health concerns. A social worker was consulted if there was a failed screen. The PHQ-9 scores range from 0 to 27. Scores of 5–9 are classified as mild depression; 10–14 as moderate depression; 15–19 as moderately severe depression and ≥ 20 as severe depression.

#### Statistical analysis

Statistical analysis was conducted in SAS 9.0. Differences in proportions were assessed by Fisher exact tests, and factors influencing continuous variables (HbA1c, CGM time in range, PHQ9 score) were identified using generalized linear models with age, gender, year, insurance status, race/ethnicity and (for HbA1c) CGM use as main effects. Estimates were not adjusted for additional covariates.

We retrospectively assessed whether a previously-developed model of hospitalization risk [6] retained discrimination under the changed circumstances of the pandemic.

## Results

For all results, the “2020” group represents encounters occurring between 3/15/2020 (the approximate day that COVID restrictions were imposed in Dallas County) and 3/14/2021; the “2019” group comprises encounters between 3/15/2019 and 3/14/2020. In 2020, the mean age was 13.8 ± 3.6 years; 52.8% were male; 53.3% were White, 22.3% Hispanic and 17.7% Black; 60.3% had commercial insurance.

**Fig. 1:**
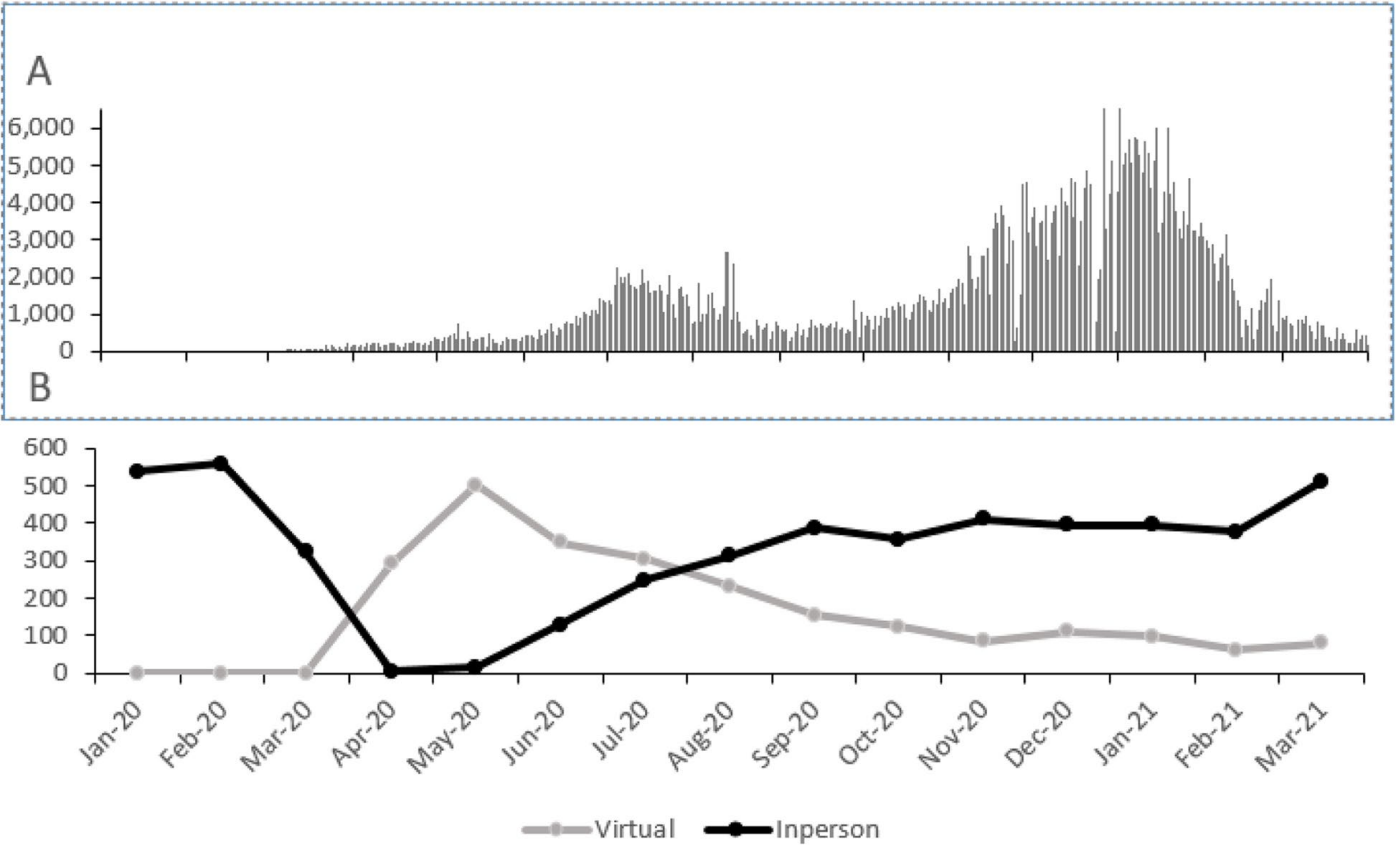
Utilization of virtual visits in relation to the COVID pandemic. Panel **A** shows COVID cases in the Dallas Forth-Worth area and Panel **B** shows diabetes clinic visits (virtual and in person) from 1/2020 to 3/2021)

In response to the pandemic, all clinic visits were suspended on 3/16/2020 and a virtual visit platform was quickly put in place starting on 4/1/2020. Our operations gradually transitioned back to clinic visits starting on 5/1/2020 and gradually ramped up. (Fig. 1).

In 2020, the number of outpatient visits (including both clinic and virtual visits) per patient decreased markedly for those with commercial insurance but there was not a significant decrease per patient with non-commercial insurance (Table 1). Patients with commercial insurance had fewer office visits per patient than those with non-commercial insurance. However, there was no difference in utilization of the virtual visit platform in patients based on insurance status.

**Table 1:** Outpatient visit frequency

		**Visits**					
		**0**	**1**	**2**	** > 3**	**Total**	***P***^**+**^
**Count of patients with each number of total outpatient visits, by year**^**a**^							
**Commercial**							
**2019**		–	150	254	649	1053	
	**%**	–	14.2	24.1	61.6	100	
**2020**		–	252	331	401	984	
	**%**	–	25.6	33.6	40.8	100	
**Total**		–	402	585	1050	2037	< 0.0001
**Non-commercial**							
**2019**		–	139	172	382	693	
	**%**	–	20.0	24.8	55.1	100	
**2020**		–	150	179	318	647	
	**%**	–	23.2	27.7	49.1	100	
**Total**		–	289	351	700	1340	0.09
**Count of patients with each number of office and virtual visits in 2020**							
**Office**							
**Commercial**		262	302	264	156	984	
	**%**	26.6	30.7	26.8	15.9	100	
**Non-commercial**		136	197	181	133	647	
	**%**	21.0	30.4	28.0	20.6	100	
**Total**		398	499	445	289	1631	0.02
**Virtual**							
**Commercial**		363	391	168	62	984	
	**%**	36.9	39.7	16.5	6.6	100	
**Non-commercial**		240	251	107	49	647	
	**%**	37.1	38.8	16.5	7.6	100	
**Total**		603	642	275	95	1631	NS

+ *P* values by Fisher Exact Tests

^a^Patients were included in the analysis for a given year only if they had at least one outpatient visit

There was no change in hospitalization frequency from 2019 to 2020 in either commercially or non-commercially insured patients (Table 2), but patients with non-commercial insurance were hospitalized at markedly higher frequencies (*p* < 0.0001) in both years. The data in the Table are for both DKA (ICD10 codes of E10.10 and E10.11) and hyperglycemia (E10.65), but the hospitalization frequency was also unchanged if DKA alone was considered (271 admissions in 2019, 270 in 2020).

**Table 2:** Count of patients with each number of admissions, by year

**Commercial**		**Admissions**				
		**0**	**1**	** > 2**	**Total**	***P***
**2019**		992	47	14	1053	
	**%**	94.2	4.5	1.3	100	
**2020**		916	59	9	984	
	**%**	93.1	6.0	0.9	100	
**Total**		1908	106	23	2037	NS
**Non-Commercial**						
**2019**		581	82	30	693	
		83.8	11.8	4.3	100	
**2020**		543	75	29	647	
		83.9	11.6	4.5	100	
**Total**		1124	157	59	1340	NS

*P* values by Fisher Exact Tests

*P* < 0.0001 commercial vs non-commercial

Using data from October 2014 to October 2017, we had previously developed a predictive model for hospital admissions incorporating hospitalizations in the prior 12 months, HbA1c and non-commercial insurance as factors [6]. To see if the model retained discrimination (i.e., predictive power) under the changed circumstances of the pandemic, we used data from the 2019 period to predict hospitalization in the 2020 period. As assessed by the area under the receiver operator characteristic curve (ROC AUC), discrimination actually improved from 0.746 in the original training dataset [6] to 0.761 in the present study. In the original training dataset, a risk score of 0.3 had 95% specificity and 29% sensitivity to predict hospitalization; in the present study, the same threshold had 94% specificity and 32% sensitivity. Thus, model performance was essentially unchanged during the pandemic.

The effects of the pandemic on glycemic control were examined in a generalized linear model (Table 3). Increasing age, non-commercial insurance, Black and Hispanic race/ethnicity, and non-utilization of continuous glucose monitors (CGM) were all associated with higher HbA1c, but there was no difference between the 2019 and 2020 groups. In this and the other linear models, there were no significant interactions between year and any other covariate. There was no change in CGM utilization in patients with commercial insurance (61.8% in 2019 and 61.4% in 2020), but CGM utilization by patients with non-commercial insurance increased markedly from 24.5% in 2019 to 35.7% in 2020 (*p* = 0.001), probably because Texas Medicaid began approving reimbursement for CGM in April 2020. CGM percent time in range was strongly correlated with HbA1c (R^2^ = 0.49, *p* < 0.0001). Similar to the findings regarding HbA1c, time in range among patients utilizing CGM was lower in those with non-commercial insurance and in Black and Hispanic patients; it improved slightly from 2019 to 2020 (Table 4).

**Table 3:** Factors influencing hemoglobin A1c, linear model

	Estimate	Standard Error	*P*
Intercept	7.71	0.14	< 0.0001
Age,y	0.05	0.01	< 0.0001
Gender			
Male	0.00		
Female	0.11	0.06	0.06
Year			
2019	0.00		
2020	0.04	0.06	NS
Insurance			
Commercial	0.00		
Non-commercial	0.62	0.07	< 0.0001
Race/ethnicity			
White	0.00		
Black	1.31	0.09	< 0.0001
Hispanic	0.41	0.08	< 0.0001
Other	-0.10	0.12	NS
CGM use			
Yes	0.00		
No	0.87	0.07	< 0.0001

**Table 4:** Factors influencing CGM time in range (%), linear model

	Estimate	Standard Error	*P*
Intercept	45.59	2.02	< 0.0001
Age,y	-0.19	0.12	NS
Gender			
Male	0.00		
Female	-0.03	0.92	NS
Year			
2019	0.00		
2020	1.93	0.92	0.04
Insurance			
Commercial	0.00		
Non-commercial	-7.50	1.15	< 0.0001
Race/ethnicity			
White	0.00		
Black	-7.60	1.49	< 0.0001
Hispanic	-2.96	1.40	0.03
Other	1.74	1.83	NS

We routinely screen for depression in our patients 10 years of age and older using the Patient Health Questionaire-9 (PHQ-9); the proportion of screened patients in the entire clinic population decreased in 2020 from 58.5% to 41.5% (*p* < 0.0001) because we did not attempt to have patients complete the questionnaire online. Our PHQ-9 scores ranged from 0 to 21. Among those screened, the only demographic factor associated with increased scores was female gender; there was no significant change from 2019 to 2020 (Table 5).

**Table 5:** Factors influencing PHQ9, linear model

	Estimate	Standard Error	*P*
Intercept	2.03	0.57	0.0003
Age	0.00	0.03	NS
Year			
2019	0.00		
2020	-0.20	0.17	NS
Insurance			
Commercial			
Non-commercial	0.19	0.19	NS
Gender			
Male	0.00		
Female	0.74	0.17	< 0.0001
Race/ethnicity			
White	0.00		
Black	0.20	0.24	NS
Hispanic	0.13	0.22	NS
Other	-0.20	0.36	NS

## Discussion

The COVID-19 pandemic has had a worldwide impact on glycemic control in diabetic patients. The International Society for Pediatric and Adolescent Diabetes (ISPAD) recommended maintaining good glycemic control as an effective strategy for preventing severe COVID-19 disease and death in this population. Higher HbA1c, minority race or ethnicity, and non-commercial insurance status are independently associated with increased rates of diabetic ketoacidosis in pediatric patients with T1D [6], indicating that these subgroups may be particularly vulnerable during the current pandemic. The relatively high rates of COVID-19 in racial and ethnic minority communities may further increase their risk of poor outcomes [8]. Insulin pumps and continuous glucose monitoring (CGM) use may improve glycemic control, but minority and economically disadvantaged populations have lower use of these devices [9]. The pandemic has highlighted disparities in care for this population, who had already encountered barriers to access of care prior to the pandemic.

A review of 238 children in Alabama during the pandemic showed worsening glycemic control in children with Type 1 diabetes, with those on public insurance affected in greater proportion than those with private insurance [10]. In both India [11] and Saudi Arabia [12], difficulties with obtaining insulin during the pandemic may have affected glycemic control. Conversely, use of advanced devices such as continuous glucose monitors and closed-loop insulin pumps during the pandemic was associated with stable or improved glycemic control in many locales including China [13], Italy [14–16], Greece [17] and Israel [18].

Adoption of telemedicine visits may improve outcomes in diabetic patients; retrospective data during the COVID-19 pandemic in 2727 Japanese diabetes patients showed both telemedicine and clinic visits improved glucose control [19]. However, in a report of 28,977 patient visits over 2.5 years at a single US center, the odds of completing a visit via telemedicine were lower among non-English speaking and Medicaid insured pediatric patients. Socioeconomic status, affordability of internet services and cellular data, housing insecurity, loss of jobs, and unpredictable work schedules likely contributed to decreased uptake of telemedicine in this population [20].

In our large urban hospital setting, the COVID pandemic had no effect on glycemic control. CGM utilization increased in the non- commercial group during the pandemic due to improved coverage and efforts of our group to promote CGM access. Hospitalization frequencies were unchanged by the pandemic despite a decrease in office visits among patients with commercial insurance.

Rapid adoption of telemedicine, improved access to CGM and, perhaps, increased parental oversight of diabetes care likely helped maintain glycemic control and hospitalization frequencies at pre-pandemic levels [13].

### Strengths and limitations

The strengths of this study are the large sample size—over 1600 patients– and the inclusion of an ethnically and socioeconomically diverse population. Moreover, there are very few other hospitals in the North Texas region that routinely admit children with DKA or hyperglycemia. This minimizes referral biases and renders the present study essentially population-based (although certainly some hospitalizations may occur elsewhere and not be ascertained). On the other hand, our observations are limited to one center. We serve an urban population and our findings regarding adoption of telemedicine cannot be extrapolated to rural areas where there may have been issues with internet and cellular connectivity. Other than the PHQ-9, we have no before-and-after survey data on factors that might mediate pandemic-related changes in glycemic control such as quality of life, health habits, parental work history, physical activity or school attendance. The most recent HbA1c for each patient in each study period was the only value captured in the database; given that the number of clinic visits (and, thus, HbA1c tests) differed between the two study periods, we felt that this approach would minimize any biases arising from this disparity. Finally, we used insurance status as a surrogate for socio-economic status, although this is an imperfect measure [7].

## Conclusions

Hospitalization frequency, glycemic control and incidence of depression were unchanged among children with type 1 diabetes in a large urban children’s hospital during the first year of the COVID-19 pandemic. Increased adoption of continuous glucose monitors and extensive use of telemedicine visits may have ameliorated the impact of the pandemic on disease management.

## Data Availability

The datasets during and/or analyzed during the current study is available from the corresponding author on reasonable request.
